# Two New Guaiane Sesquiterpenoids from *Daphne holosericea* (Diels) Hamaya

**DOI:** 10.3390/molecules190914266

**Published:** 2014-09-11

**Authors:** Qing-Yun Ma, Yi-Chun Chen, Sheng-Zhuo Huang, Zhi-Kai Guo, Hao-Fu Dai, Yan Hua, You-Xing Zhao

**Affiliations:** 1 Key Laboratory of Biology and Genetic Resources of Tropical Crops, Ministry of Agriculture, Institute of Tropical Bioscience and Biotechnology, Chinese Academy of Tropical Agricultural Sciences, Haikou 571101, Hainan, China; E-Mails: maqingyun@itbb.org.cn (Q.-Y.M.); huangshengzhuo@itbb.org.cn (S.-Z.H.); guozhikai@itbb.org.cn (Z.-K.G.); daihaofu@itbb.org.cn (H.-F.D.); 2 College of Forestry, Southwest Forestry University, Kunming 650224, Yunnan, China; E-Mail: amony2009@163.com

**Keywords:** Thymelaeaceae, *Daphne holosericea*, guaiane sesquiterpenoids, acetylcholinesterase inhibition

## Abstract

Two new sesquiterpenoids with guaiane skeletons—holosericin A (**1**) and holosericin B (**2**)—were isolated from the medicinal plant *Daphne holosericea* (Diels) Hamawa (Thymelaeceae). Their structures were elucidated by 1D and 2D-NMR spectroscopy, as well as HR-ESI-MS data. Compounds **1** and **2** were evaluated for inhibitory activities against acetylcholinesterase and compound **2** showed a moderate activity with 31% inhibition.

## 1. Introduction

**Figure 1:**
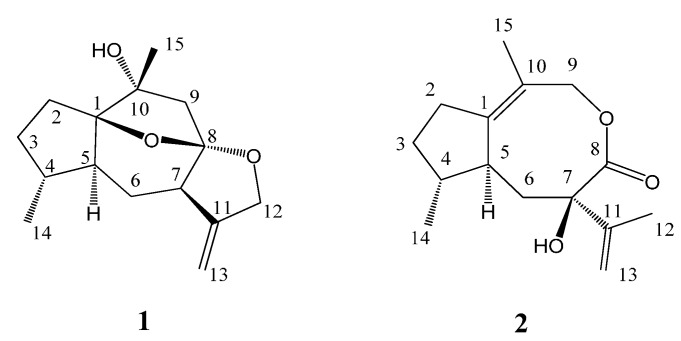
Structures of compounds **1**–**2**.

*Daphne* is an important genus of the family Thymelaeaceae widely distributed around the world, and among which some species are used as traditional Chinese medicine [1]. Phytochemical investigations on some common *Daphne* species showed that the main type of natural products present were terpenoids, including sesquiterpenoids and diterpenoids [2,3,4,5,6,7] with extensive pharmacological activities as antitumor [8], antifeedant [2], antiinflammation [6], and anti-HIV-1 agents [7,9]. *Daphne holosericea* (Diels) Hamawa plants grow in the wet valleys of southwestern China at 2000 m above sea level [10] and its limited chemical studies have reported the presence of flavonoids [11] and phenylpropanoids [12]. In the course of our systematic chemical research on the genus *Daphe* [2,5,7,9] for the search for new bioactive constituents, a chemical investigation on *D. holosericea* have been carried out and two new sesquiterpenoids with unusual guaiane skeletons, holosericin A (**1**) and holosericin B (**2**) (Figure 1), were isolated from the EtOAc extract of this plant. Herein, we describe the isolation, structural elucidation of the new compounds, as well as their acetylcholinesterase (AChE) inhibitory activities.

## 2. Results and Discussion

**Figure 2:**
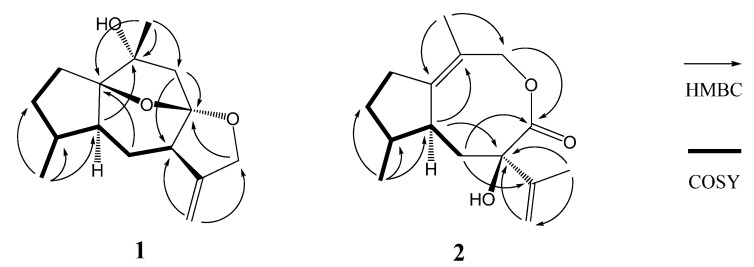
Key HMBC and COSY correlations of compounds **1** and **2**.

Compound **1** was obtained as a colorless oil, and its molecular formula was assigned to be C_15_H_22_O_3_ from its HR-EI-MS (*m/z* 250.1567 [M]^+^) and NMR data (Table 1), indicating five degrees of unsaturation. The IR spectrum revealed the presence of hydroxyl (3417 cm^−1^) and double bond (1597 cm^−1^) absorptions. The ^1^H-NMR spectrum (Table 1) of compound **1** exhibited signals of two methyl [δ_H_ 1.38 (3H, s, H-15) and 0.96 (3H, d, *J* = 6.9 Hz, H-14)], one oxygenated methylene [δ_H_ 4.56 (1H, d, *J* = 13.0 Hz, H-12a) and 4.48 (1H, d, *J* = 13.0 Hz, H-12b)], and one terminal olefinic bond [δ_H_ 4.95 (1H, d, *J* = 2.4 Hz, H-13a) and 4.93 (1H, d, *J* = 2.4 Hz, H-13b)]. The ^13^C-NMR (DEPT) spectroscopic data (Table 1) showed the presence of 15 carbon resonances for two methyl, six methylene (one oxygenated and one olefinic), three methine, and four quaternary carbons (one olefinic and three oxygenated), indicative of a possible sesquiterpenoid skeleton. Further comprehensive analysis of the 1D- and 2D-NMR spectra indicated that compound **1** had the same guaiane skeleton as 4β,10α-dihydroxy-5α(H)-1,11(13)-guaidien-8α,12-olide [13]. The differences between two compounds were one oxygenated quaternary carbon (C-1), one saturated methylene (C-2) and one methane (C-4) in compound **1** replacing the corresponding double bond and the oxygenated quaternary carbon in 4β,10α-dihydroxy-5α(H)-1,11(13)-guaidien-8α,12-olide. This was further proved by the ^1^H ^1^H COSY correlations of H-2/H-3, H-3/H-4, H-4/H-14, H-4/H-5, H-5/H-6, and H-6/H-7 (Figure 2), indicating the presence of the partial structures C-2-C-3-C-4(-C-14)-C-5-C-6-C-7, and HMBC correlations from H-14 to C-4 (δ_C_ 38.3) and from H-15 to C-1 (δ_C_ 93.4) and C-10 (δ_C_ 76.1) (Figure 2). The other difference was the oxygenated methylene (C-12) replacing the carbonyl to firm the epoxy group at C-8 and C-12 in compound **1**, which was confirmed by HMBC correlations from H-13 to C-12 (δ_C_ 72.2) and from H-12 to C-8 (δ_C_ 110.2). The additional epoxy group between C-8 and C-1 in compound **1** was deduced based on the requirement of degrees of unsaturation, the molecular model as well as the reasonable chemical shifts of C-8 and C-1. The planar structure of compound **1**, a 5/6/5/5 ring system via two oxygen bridges, was established and its stereochemistry needed to be further confirmed.

**Table 1:** ^1^H- and ^13^C-NMR data for compounds **1** and **2** (CDCl_3_).

No.	1		2	
	δ_C_	δ_H_, * J* (Hz)	δ_C_	δ_H_, * J* (Hz)
1	93.4		157.0	
2	32.1	1.74 m, 1.61 m (α-H)	39.7	1.86 m (α-H), 1.69 m
3	31.8	1.60 m, 1.45 m (α-H)	32.4	1.85 m, 1.55 m (α-H)
4	38.3	1.96 m	36.0	2.25 m
5	39.1	2.38 ddd (5.0, 8.5, 13.5)	45.5	2.46 m
6	24.9	1.82 m (α-H), 1.21 m	19.5	2.40 dd (14.0, 9.5, α-H), 2.28 dd (14.0, 6.0)
7	49.8	2.50 dd (10.5, 8.0)	84.7	
8	110.2		176.5	
9	50.6	2.22 d (13.6), 2.03 d (13.6, α-H)	72.8	4.56 s
10	76.1		126.9	
11	152.1		149.9	
12	72.2	4.48 d (13.0, α-H), 4.56 d (13.0)	19.6	1.67 s
13	104.6	4.95 d (2.4), 4.93 d (2.4)	109.3	4.90 s, 4.72 s
14	14.6	0.96 d (6.9)	17.5	1.06 d (7.5)
15	26.5	1.38 s	12.5	2.02 s

**Figure 3:**
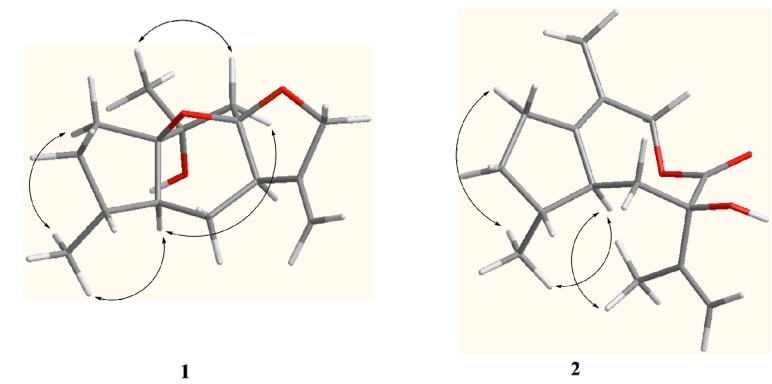
Key ROESY correlations of compounds **1** and **2**.

The relative configuration of compound **1** was proposed based on a ROESY experiment (Figure 3). The ROESY correlation of CH_3_-14 with H-5 (δ_H_ 2.38, ddd, *J* = 13.5, 8.5, 5.0 Hz) revealed that H-5 and CH_3_-14 were located at the same side and assumed to be α-orientation. The α-orientation of H-7 was determined from NOE of H-5/H-9α (δ_H_ 2.03, d, *J* = 13.6 Hz) and H-7/H-9α. The β-orientation of CH_3_-15 was deduced by the key NOE of CH_3_-15/H-9β (δ_H_ 2.22, d, *J* = 13.6 Hz). Moreover, the NOE of H-5/H-9α also indicated that the partial structure C-9-C-10 connected to C-1 was α-oriented in the five number (C-1-C-5) ring system and the 1,8-epoxy was accordingly β-oriented, which correspondingly assigned the α-orientation of 8,12-epoxy. Based on above analysis, compound **1** was elucidated as 10α*-*hydroxyl-1(8),8(12)-diepoxy-11(13)-guaiene and named holosericin A.

Compound **2**, obtained as a colorless oil, had the same molecular formula C_15_H_22_O_3_ as **1** based on its HR-EI-MS (*m/z* 250.1575 [M]^+^), with five degrees of unsaturation. The IR spectrum also displayed the presence of hydroxyl (3398 cm^−1^), carbonyl (1643 cm^−1^) and olefinic bonds (1597 cm^−1^) absorptions. The ^1^H-NMR spectrum (Table 1) of compound **2** showed signals of three methyl [δ_H_ 2.20 (3H, s, H-15), 1.67 (3H, s, H-12) and 1.06 (3H, d, *J* = 7.5 Hz, H-14)] and one terminal olefinic bond [δ_H_ 4.90 (1H, s, H-13a) and 4.72 (1H, s, H-13b)]. The ^13^C-NMR (DEPT) spectra (Table 1) showed the presence of 15 carbon resonances for three methyl, five methylene, two methine, and five quaternary carbons, suggestive of a sesquiterpenoid skeleton. The assignment of compound **2** was based on the comprehensive analysis of the 1D and 2D-NMR spectroscopic data including ^1^H-^1^H COSY, HSQC, and HMBC spectra. The ^1^H-^1^H COSY correlations of H-2/H-3, H-3/H-4, H-4/H-14, H-4/H-5, and H-5/H-6 (Figure 2) indicated the presence of the partial structures C-2-C-3-C-4(-C-14)-C-5-C-6. The detail HMBC correlations (Figure 2) indicated the structure of compound **2** possessed the skeleton of 8,9-seco guaiane. The key HMBC correlation from H-9 [δ_H_ 4.56 (s)] to C-8 (δ_C_ 176.5) determined the presence of 8,9-olide group in compound **2**. Thus, the planar structure of compound **2**, a 5/8 ring system, was established as shown in Figure 1. The relative configurations of the stereogenic centers (C-4, C-5, and C-7) of compound **2** were determined by the ROESY experiment. The key ROESY correlation of H-5 (δ_H_ 2.46, m) with CH_3_-14 indicated that H-5 and CH_3_-14 were located at the same side and assumed to be α-orientation. The β-orientation of 7-OH was assigned from the NOE of H-5/CH_3_-12. Based on above evidence, compound **2** was elucidated as 8,9-seco-1(10),11(13)-guaidien-8,9-olide and named holosericin B.

The AChE inhibitory activities of compounds **1** and **2** were determined according to the previously described method [14,15]. The known AChE inhibitor tacrine was used as positive control in this assay and showed the percentage inhibition of 57%. Compound **2** exhibited certain inhibitory activity with a 31% inhibition at the concentration of 100 µmol∙L^−1^, whereas compound **1** showed no activity.

## 3. Experimental Section

### 3.1. General Information

Optical rotations were recorded using a Rudolph Autopol III polarimeter (Rudolph, Hackettstown, NJ, USA). The UV spectra were measured on a Beckman DU800 spectrometer (Beckman, Brea, CA, USA). The IR spectra were obtained as KBr pellets on a Nicolet 380 FT-IR instrument (Thermo, Pittsburgh, PA, USA). The NMR spectra were recorded on a Bruker AV-500 spectrometer (Bruker, Bremen, Germany), using TMS as an internal standard. ESI-MS and HR-EI-MS were measured with an API QSTAR Pulsar 1 spectrometer (Bruker). Column chromatography was performed with silica gel (Qingdao Marine Chemical Industry Factory, Qingdao, China) and Sephadex LH-20 (Merck, Darmstadt, Germany). TLC was performed with silica gel GF_254_ (Qingdao Marine Chemical Industry Factory). Fractions were monitored by TLC and spots were visualized by heating after spraying with 5% H_2_SO_4_ in ethanol.

### 3.2. Plant Material

The stems of *Daphne holosericea* (Diels) Hamawa were collected in Deqin, Yunnan Province, People’s Republic of China. A voucher specimen (HUANG0010) identified by Y. Niu (Kunming Institute of Botany, Chinese Academy of Sciences) was deposited at the Institute of Tropical Bioscience and Biotechnology, Chinese Academy of Tropical Agricultural Sciences.

### 3.3. Extraction and the Isolation

The dry stems of *D. holosericea* (2.0 kg) were powdered and extracted with EtOH (95%) under reflux for three times (3 × 6 L). The combined extracted EtOH solution was evaporated under reduced pressure, then suspended in water and partitioned successively with EtOAc (3 × 4 L) and *n*-BuOH (3 × 4 L). The EtOAc extract (192 g) was separated by silica gel column using a gradient solvent petroleum ether/EtOAc (8:1–1:2 10 L) to afford fractions A/E. Fraction B (16.3 g) was separated by silica gel column chromatography (CC) using a gradient solvent petroleum ether/EtOAc (15:1–1:1 8 L) to afford fractions B1-B4. Fr. B2 (2.1 g) was purified over Sephadex LH-20 column (CHCl_3_/MeOH 1:1 3 L) to give two subfractions B2-1 and B2-2. Subfr.B2-1 (300 mg) was subjected to repeated RP-18 column (MeOH/H_2_O 2:1 2 L) and was further purified by repeated silica gel CC with petroleum ether/acetone (5:1, 1.5 L) and Sephadex LH-20 (CHCl_3_/MeOH 1:1 2 L) to yield **1** (4.0 mg) and **2** (6.5 mg).

*Holosericin A* (**1**). Colorless oil; C_15_H_22_O_3_, 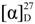 + 6.7 (c 0.5, MeOH); UV (MeOH) λ_max_ (logε) 203 (3.26); IR (KBr) ν_max_ 3417, 2924, 2852, 1597, 1420, 1083, 1032; ^1^H- and ^13^C-NMR data see Table 1; positive ESI-MS *m/z* [M+Na]^+^ 273 (100); HR-EI-MS *m/z* [M]^+^ 250.1567 (calcd. for C_15_H_22_O_3_, 150.1569).

*Holosericin B* (**2**). Colorless oil; C_15_H_22_O_3_, 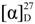 + 5.2 (c 0.5, MeOH); UV (MeOH) λ_max_ (logε) 217 (3.76), 204 (3.73); IR (KBr) ν_max_ 3398, 2922, 2852, 1643, 1597, 1419, 1042; ^1^H- and ^13^C-NMR data see Table 1; positive ESI-MS *m/z* [M+Na]^+^ 273 (60); HR-EI-MS *m/z* [M]^+^ 250.1575 (calcd. for C_15_H_22_O_3_, 150.1569).

### 3.4. Bioassay of AChE Inhibitory Activity

AChE inhibitory activity of these compounds was assayed by the spectrophotometric method [14,15]. Acetylthiocholine iodide (Sigma, St. Louis, MO, USA) was used as substrate in the assay. Compounds were dissolved in dimethyl sulfoxide (DMSO). The reaction mixture, consisting of 110 µL phosphate buffer (pH 8.0), 10 µL of tested compounds solution (2000 µmol·L^−1^), and 40 µL AChE solution (0.04 U/100 µL), was mixed and incubated for 20 min (30 °C). The reaction was initiated by the addition of 20 µL 5,5-dithiobis-2-nitrobenzoic acid (6.25 mmol·L^−1^) and 20 µL acetylthiocholine. The hydrolysis of acetylthiocholine was monitored at 405 nm after 30 min. Tacrine (Sigma-Aldrich 99%, Milwaukee, WI, USA) was used as positive control. All the reactions were done in triplicate. The percentage inhibition was calculated as follows: % inhibition = (*E* − *S*)/*E* × 100 (*E* is the activity of the enzyme without test compound and *S* is the activity of enzyme with test compounds).

## 4. Conclusions

The chemical investigation of *Daphne holosericea* led to the isolation of two new sesquiterpenoids with guaiane skeletons, holosericin A (**1**) and holosericin B (**2**). Holosericin A possessed a 5/6/5/5 ring system via two oxygen bridges and holosericin B was a 8,9-seco one. Evaluation of the inhibitory activities against acetylcholinesterase of the two new compounds showed holosericin B exhibited a moderate activity with 31% inhibition.

## Supplementary Materials

Supplementary materials can be accessed at: http://www.mdpi.com/1420-3049/19/9/14266/s1.

## Supplementary Files

Supplementary File 1
