# Supplementary material for: Two New Guaiane Sesquiterpenoids from Daphne holosericea (Diels) Hamaya
Source: Molecules. 2014 Sep 11;19(9):14266–72. doi: 10.3390/molecules190914266 (PMC6271883; doi:10.3390/molecules190914266)
Supplement: Supplementary File 1 [file molecules-19-14266-s001.pdf]

Supplementary Materials

Figure S1. ^{1}H-NMR spectrum of compound **1** in $CDCl_3$.

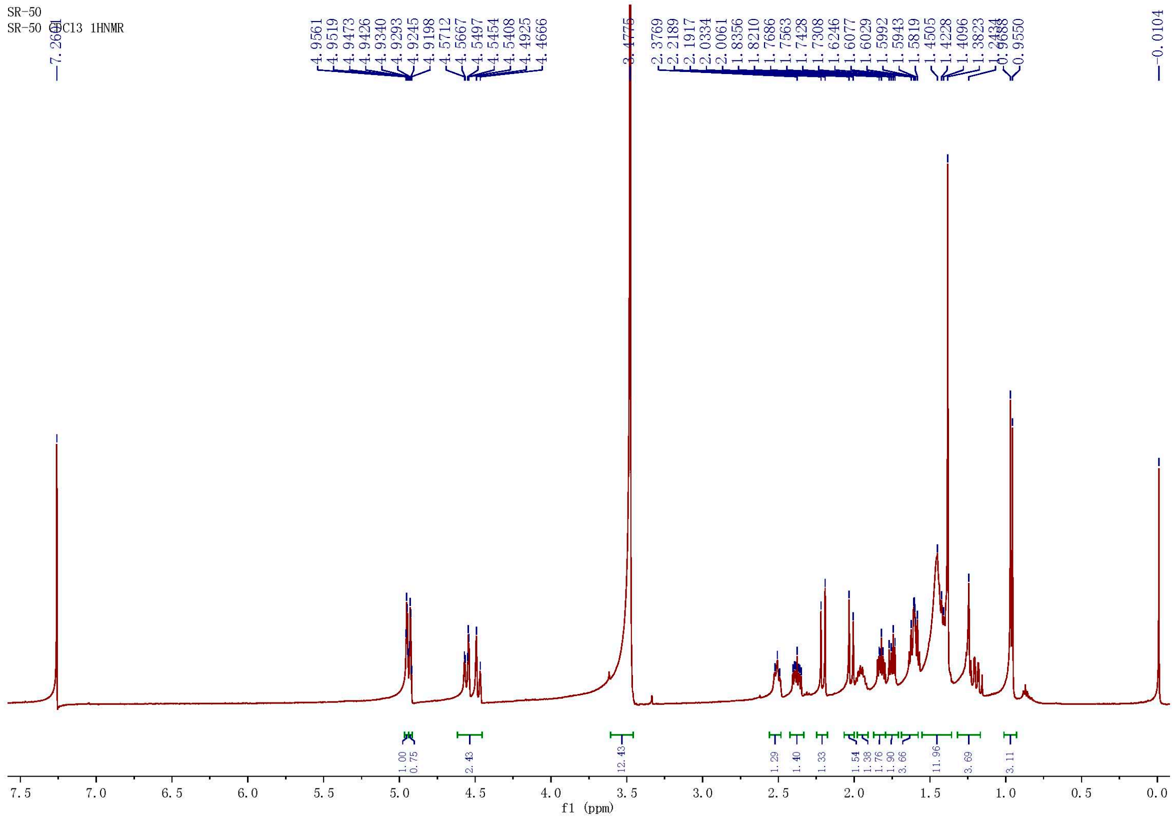

Figure S2. ^{13}C-NMR and DEPT spectrum of compound **1** in $CDCl_3$.

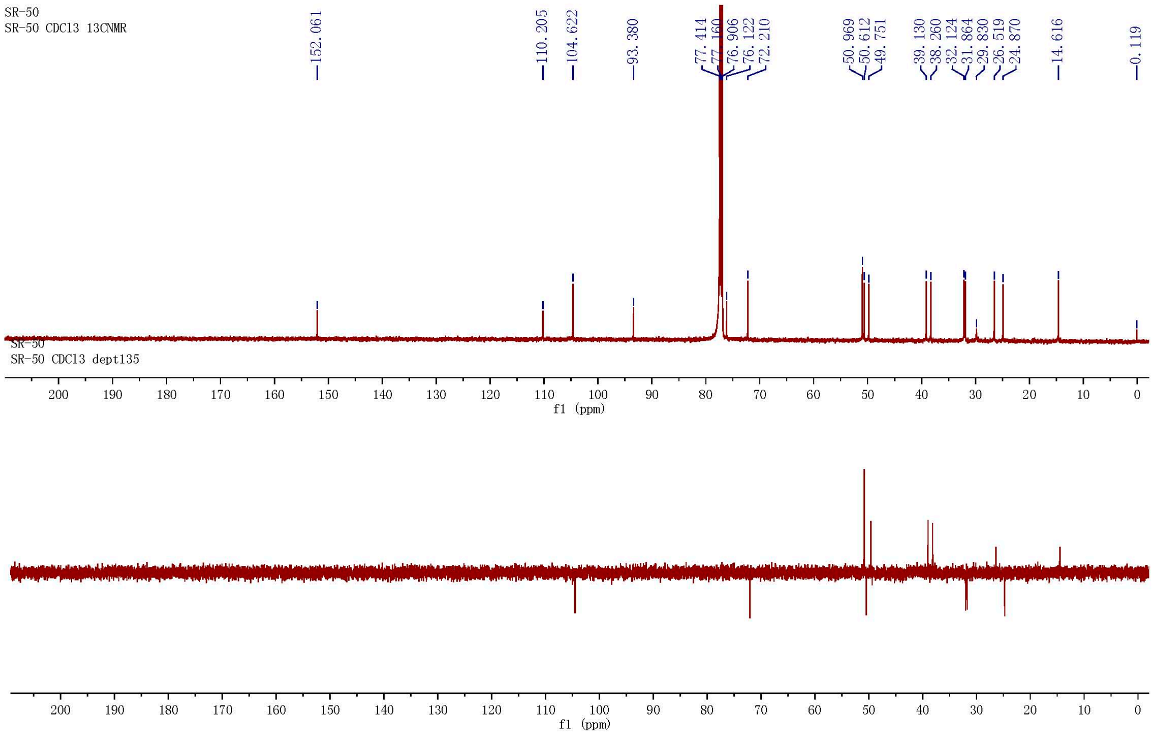

Figure S3. HSQC spectrum of compound **1** in $CDCl_3$.

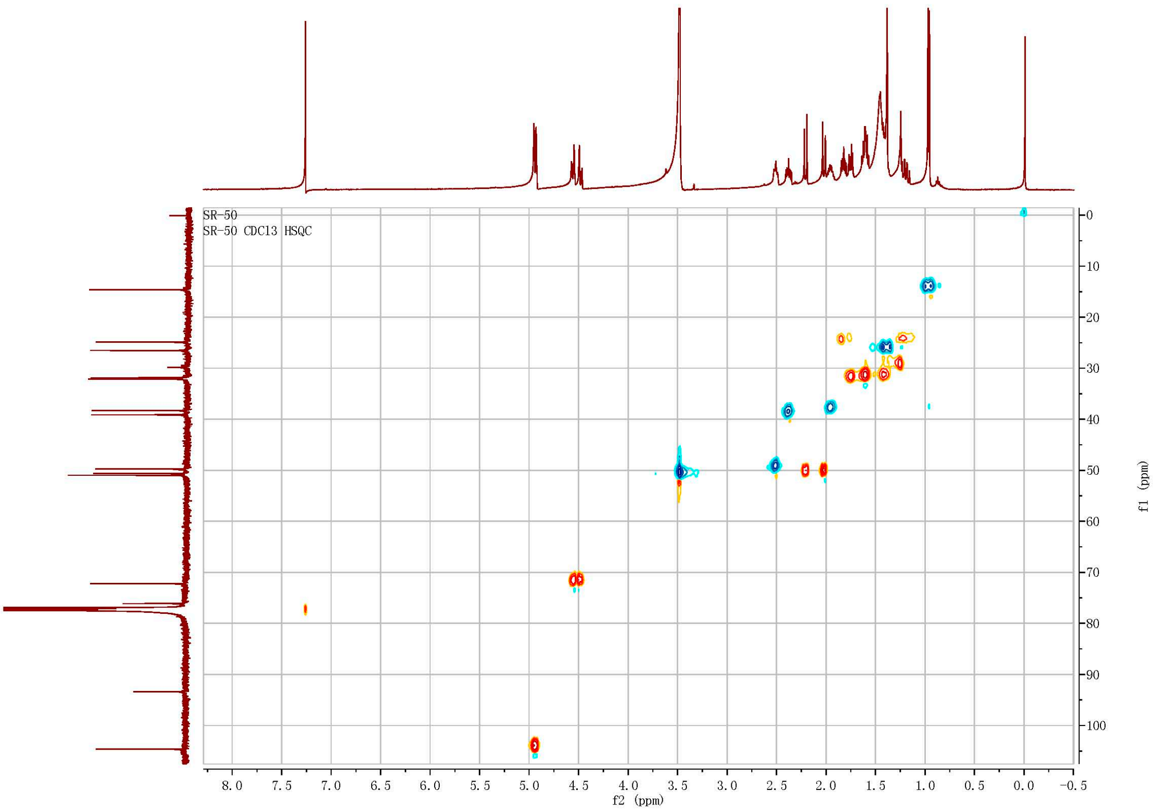

Figure S4. HMBC spectrum of compound **1** in $CDCl_3$.

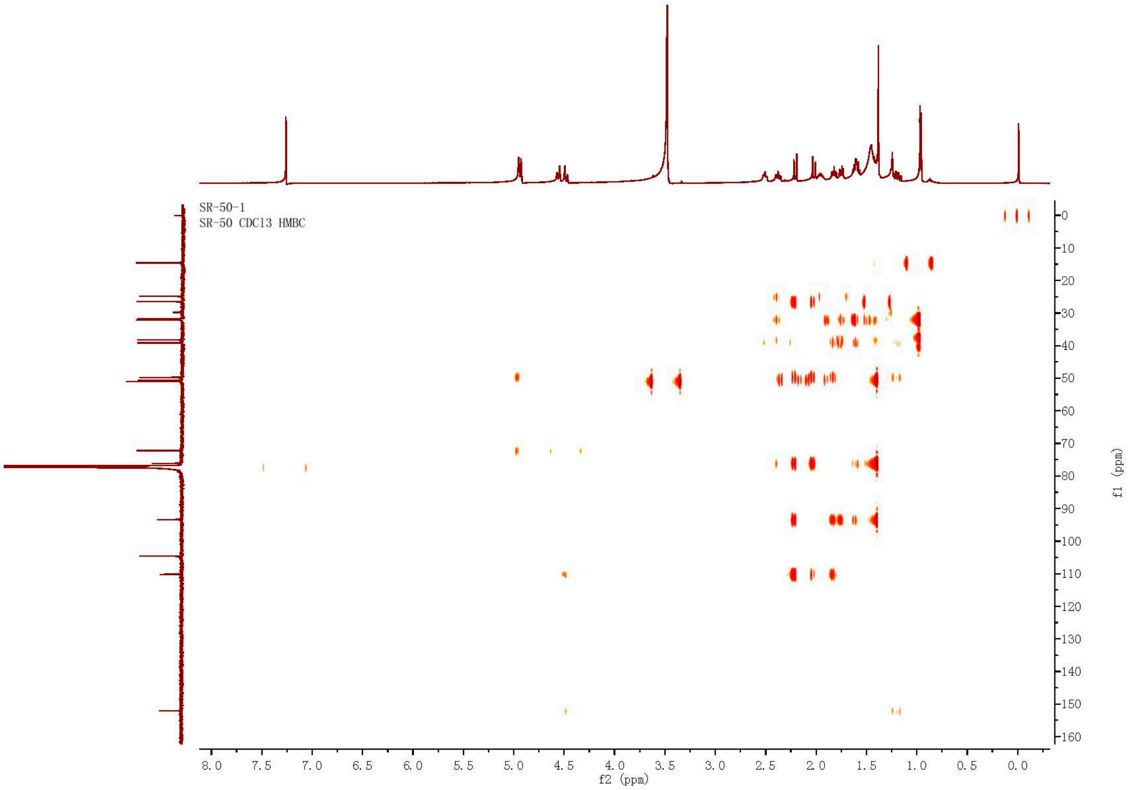

Figure S5. ^{1}H-^{1}H COSY spectrum of compound **1** in $CDCl_3$.

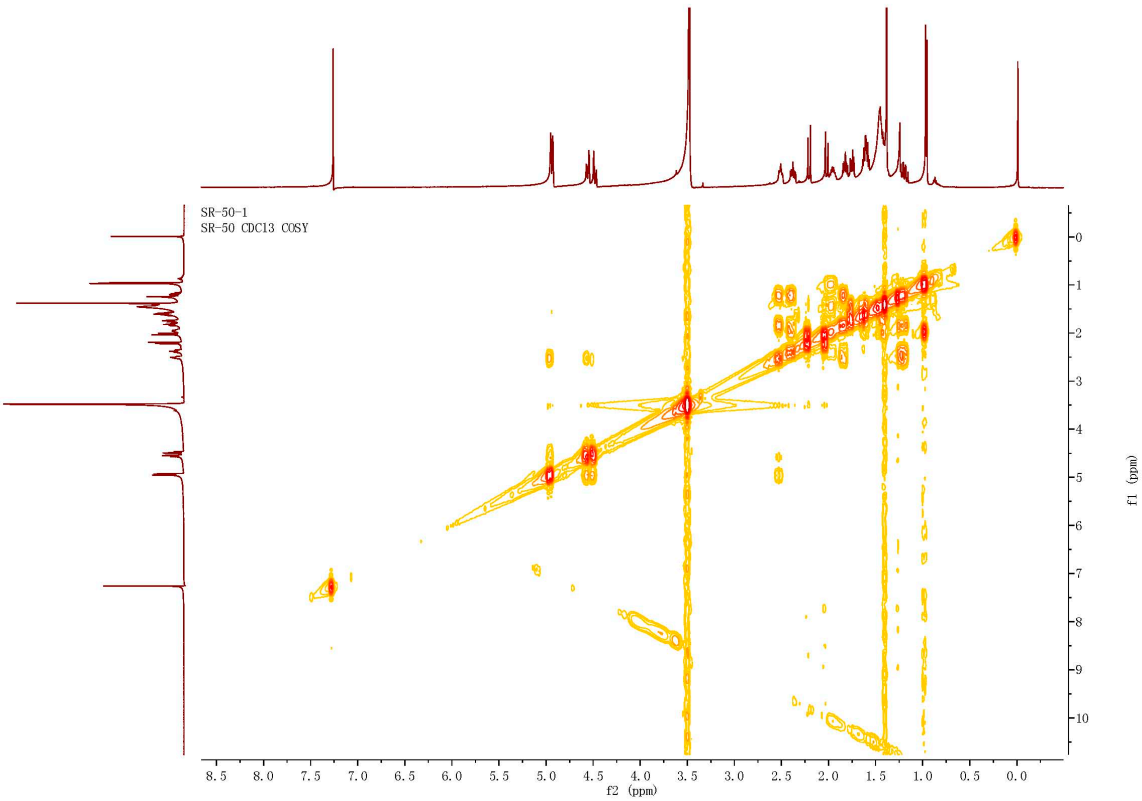

Figure S6. ROESY spectrum of compound **1** in $CDCl_3$.

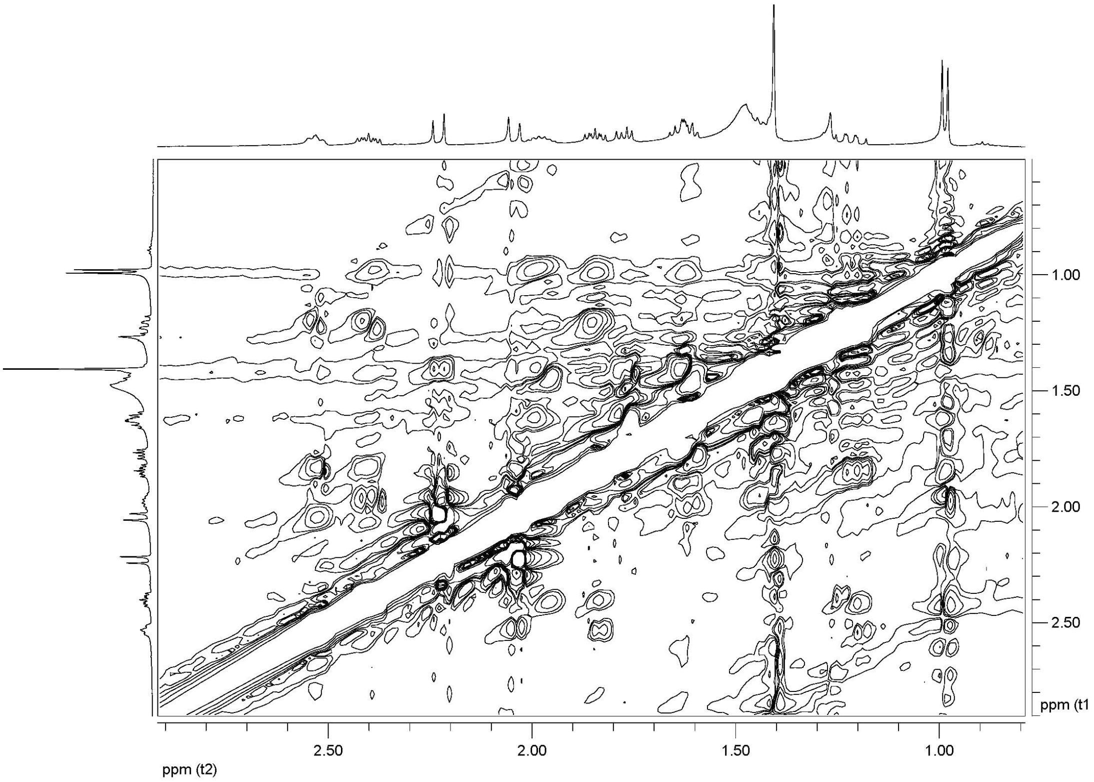

Figure S7. HREIMS spectrum of compound **1** in $CHCl_3$.

Elemental Composition Report | Page 1

Single Mass Analysis
Tolerance = 10.0 PPM / DBE: min = -10.0, max = 120.0
Selected filters: None

Monoisotopic Mass, Odd and Even Electron Ions
13 formula(e) evaluated with 1 results within limits (up to 51 closest results for each mass)
Elements Used:
C: 0-200 H: 0-400 O: 2-4

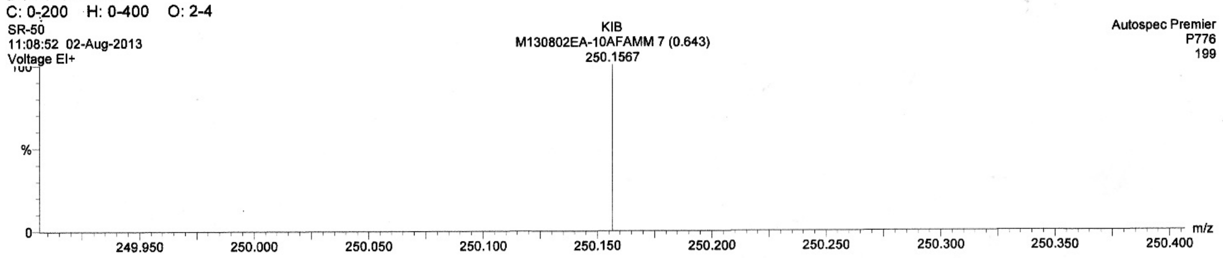

Mass	Calc. Mass	mDa	PPM	DBE	i-FIT	Formula
Minimum:				-10.0		
Maximum:		200.0	10.0	120.0		
250.1567	250.1569	-0.2	-0.8	5.0	5546105.0	C15 H22 O3

Figure S8. ^{1}H-NMR spectrum of compound **2** in $CDCl_3$.

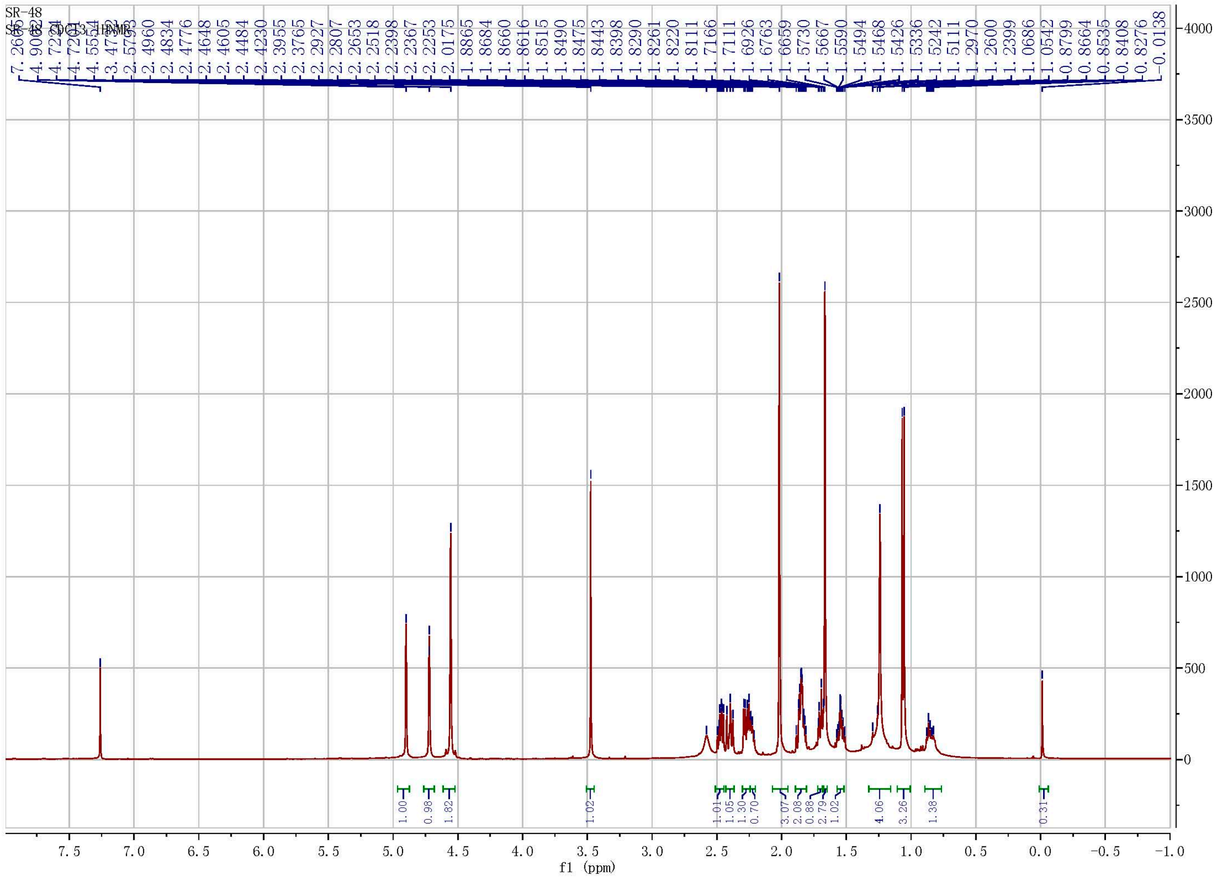

Figure S9. ^{13}C-NMR and DEPT spectrum of compound **2** in $CDCl_3$.

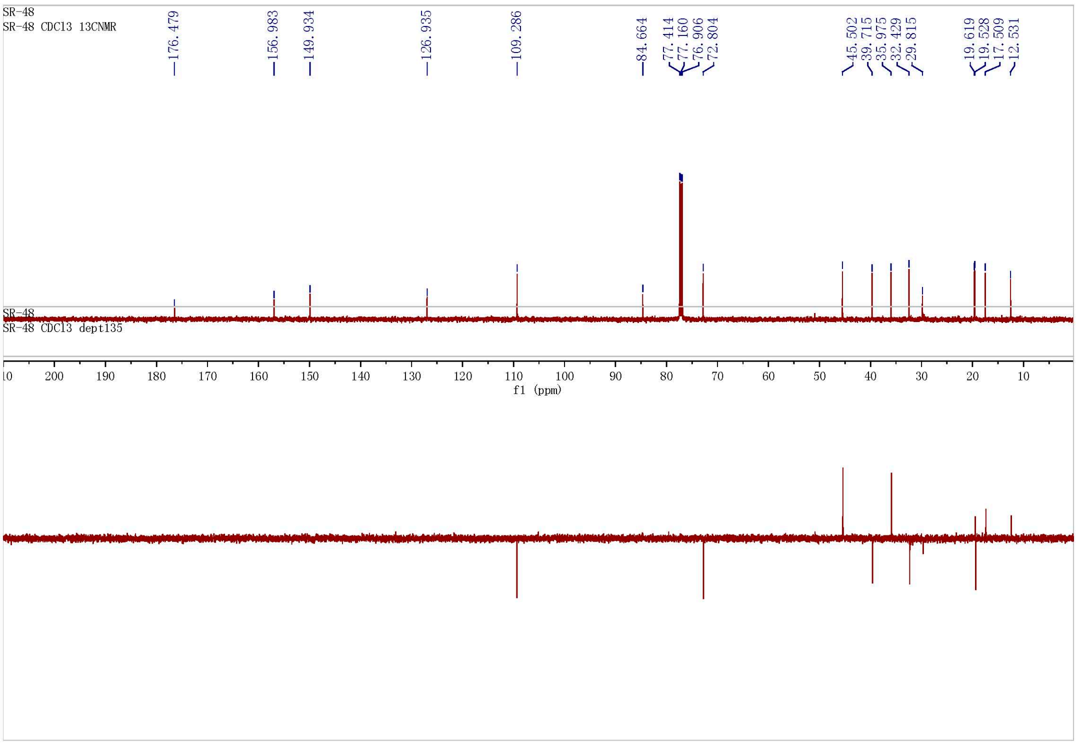

Figure S10. HSQC spectrum of compound **2** in $CDCl_3$.

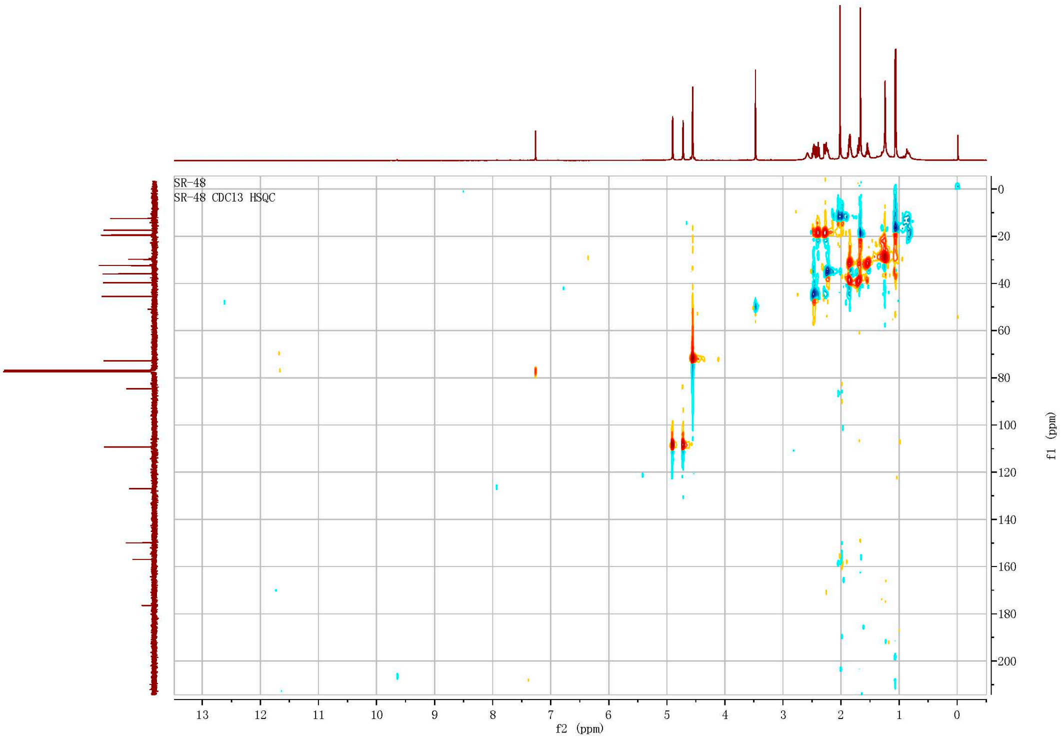

Figure S11. HMBC spectrum of compound **2** in $CDCl_3$.

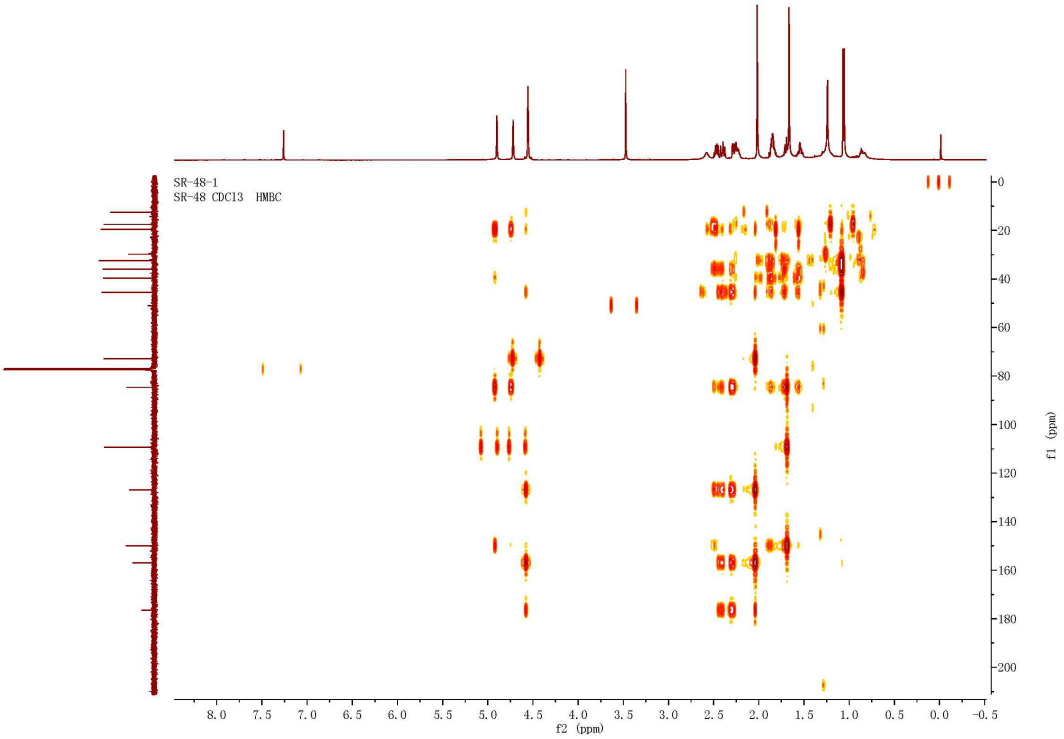

Figure S12. ^{1}H-^{1}H COSY spectrum of compound **2** in $CDCl_3$.

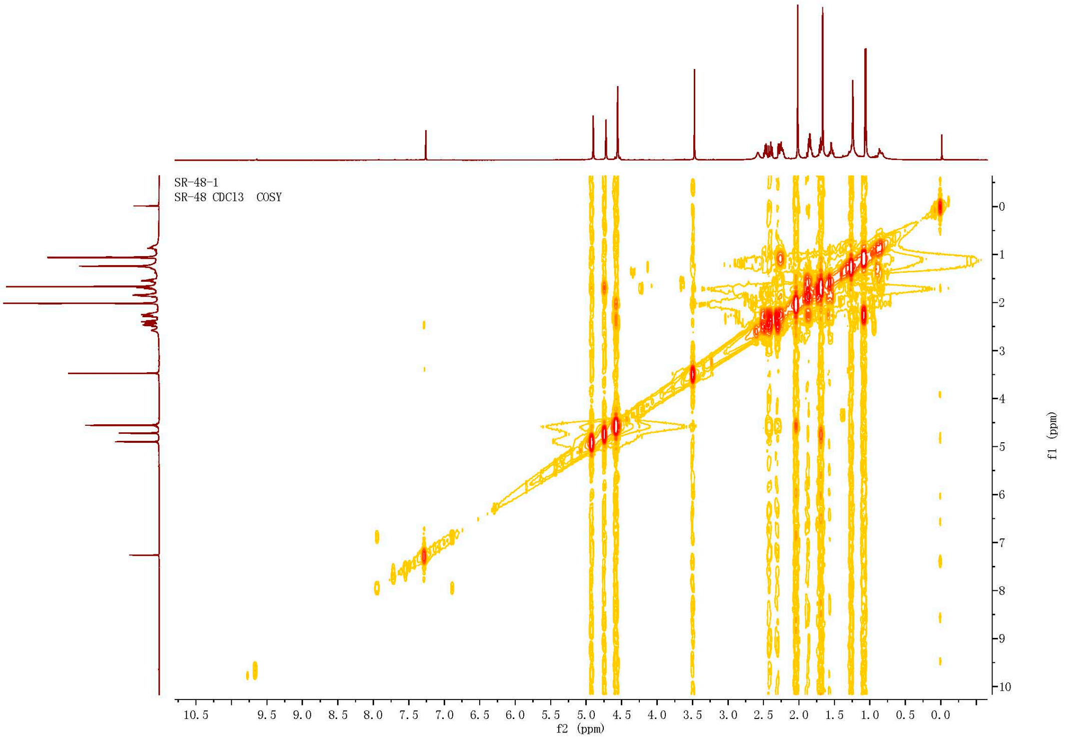

Figure S13. ROESY spectrum of compound **2** in $CDCl_3$.

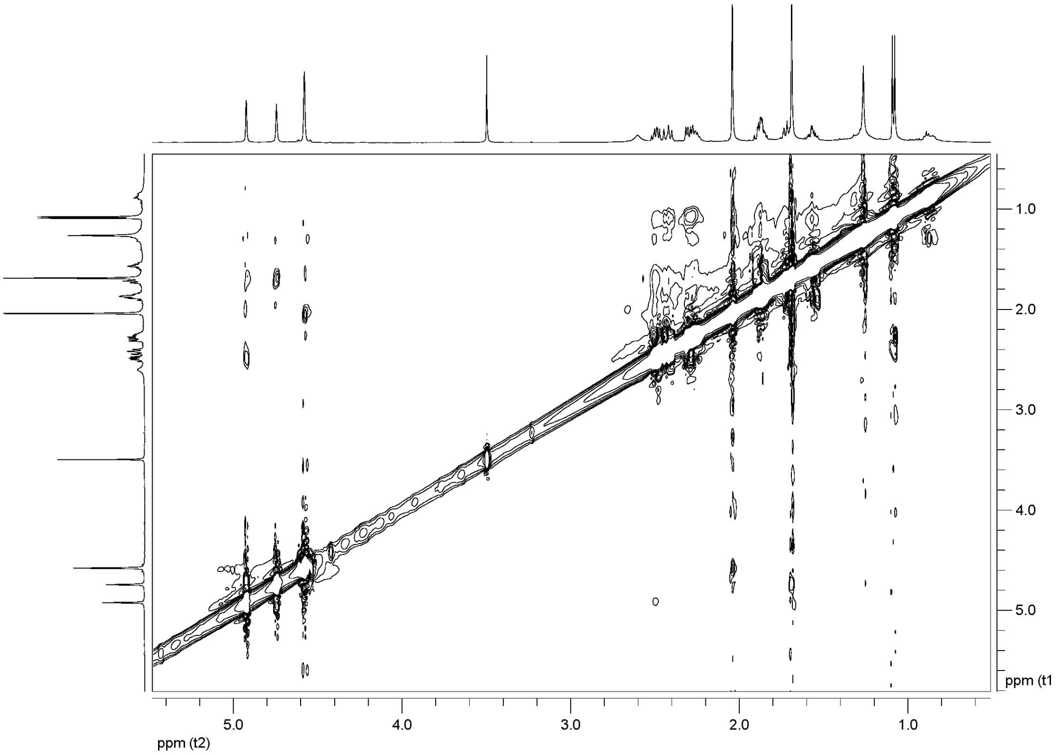

Figure S14. HREIMS spectrum of compound **2** in $CHCl_3$.

Elemental Composition Report — **Page 1**

Single Mass Analysis
Tolerance = 10.0 PPM / DBE: min = -10.0, max = 120.0
Selected filters: None

Monoisotopic Mass, Odd and Even Electron Ions
13 formula(e) evaluated with 1 results within limits (up to 51 closest results for each mass)
Elements Used:
C: 0-200 H: 0-400 O: 2-4

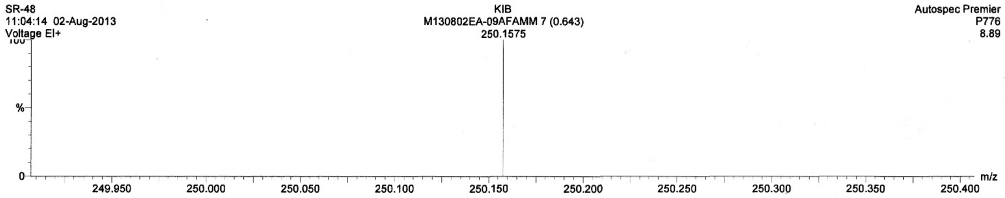

Minimum:				-10.0		
Maximum:		200.0	10.0	120.0		
Mass	Calc. Mass	mDa	PPM	DBE	i-FIT	Formula
250.1575	250.1569	0.6	2.4	5.0	5546025.5	C15 H22 O3
